# Injecting drug use in Manipur and Nagaland, Northeast India: injecting and sexual risk behaviours across age groups

**DOI:** 10.1186/1477-7517-11-27

**Published:** 2014-10-13

**Authors:** Gregory Armstrong, Amenla Nuken, Gajendra K Medhi, Jagadish Mahanta, Chumben Humtsoe, Melody Lalmuanpuaii, Michelle Kermode

**Affiliations:** Nossal Institute for Global Health, Melbourne School of Population and Global Health, University of Melbourne, Level 4, 161 Barry Street, Carlton, VIC 3010 Australia; Regional Medical Research Centre (RMRC), Indian Council of Medical Research (ICMR), Dibrugarh, Assam India; Emmanual Hospital Association, Guwahati, India

**Keywords:** Injecting drug use, HIV, Youth

## Abstract

**Background:**

There is an HIV epidemic among people who inject drugs (PWID) in Manipur and Nagaland, Northeast India. Approximately one-third of PWID across these two states are aged below 25 years, yet until now there has been no systematic investigation of the differences between the younger and older PWID. We sought to profile differences in drug use and sexual practices across age groups and to examine whether age is associated with injecting and sexual risk behaviours.

**Methods:**

We used combined cross-sectional survey data collected in 2009 from two surveys involving a total of 3,362 (male) PWID in eight districts of Manipur and Nagaland. All data were collected using interviewer-administered questionnaires.

**Results:**

Compared to PWID aged 35 years or older, PWID aged 18 to 24 years were more likely share needles/syringes in both Manipur (OR =1.8) and Nagaland (OR =1.6). Compared to PWID aged 35 years or older, PWID aged 18 to 24 years were almost two times as likely to draw up drug solution from a common container at their last injection in Manipur (OR =1.8). In Nagaland, PWID aged 18 to 24 years were more likely to use condoms consistently with both casual (OR =3.1) and paid (OR =17.7) female sexual partners compared to PWID aged 35 years or older.

**Conclusion:**

Risky injecting practices were more common among younger PWID in both Manipur and Nagaland, while unprotected sex was more common among older PWID in Nagaland. There is a clear need to focus public health messages across different age groups.

## Background

People who inject drugs (PWID) are at high risk of infection with blood-borne viruses and are targeted by HIV prevention interventions around the world [1, 2]. Approximately 10% of HIV infections worldwide are attributable to injecting drug use, and several countries in Asia are confronting serious HIV epidemics among PWID [3]. In India, risky injecting practices remain common and represent an important driver of new HIV infections [4–6]. Additionally, risky sexual behaviours are common among PWID in India generating opportunities for the transmission of HIV and sexually transmitted infections (STIs) [4, 5].

Manipur and Nagaland are two states in the northeast region of India, an ethnically distinct and geographically isolated part of the country that is characterized by substantial under-development [7]. Both of these states share a long porous border with Myanmar, one of the world’s largest producers of heroin, and injecting of opiates has been practiced since the mid-1980s [7, 8]. Approximately 2% of the population inject drugs; the most commonly injected drugs are heroin and Spasmoproxyvon (SP), the brand name for a synthetic opiate analgesic containing dextropropoxyphene, dicyclomine hydrochloride and paracetamol [9].

Manipur and Nagaland are among the states with the highest HIV prevalence in India [10], and unsafe injecting drug use has made a major contribution to the HIV epidemic in this region [11–14]. In 2009, adult HIV prevalence was estimated to be 1.4% in Manipur and 0.8% in Nagaland (compared to 0.3% at the national level) [15]. HIV prevalence among PWID (according to 2010–11 government sentinel surveillance) was estimated to be 12.9% in Manipur and 2.2% in Nagaland [10]. The HIV prevalence in Manipur is consistently higher than Nagaland. This is in part due to very different patterns of drug use in the two states. PWID in Manipur mainly inject heroin, while those in Nagaland use SP (a synthetic opioid containing dextropropoxyphene) [16]. SP is very damaging to veins, so after a few years of use, injecting is no longer possible. As SP can be taken orally, the person does not always have to inject, so less injecting takes place overall.

The response to HIV and injecting drug use in Manipur and Nagaland was historically punitive but is now guided by internationally recognized harm reduction principles [7]. The HIV prevention response is coordinated by the government through the National AIDS Control Organization (NACO) and the respective State AIDS Control Societies (SACS). Alongside this, Avahan (Bill & Melinda Gates Foundation in India) funded Project ORCHID to coordinate a range of local non-government organizations (NGOs) to implement HIV prevention interventions in selected districts of Manipur and Nagaland over a 10-year period (2004–14). Regardless of funding source, most of the targeted HIV prevention interventions in Manipur and Nagaland are delivered in the field by local NGOs. To evaluate the HIV prevention interventions, and to better understand the dynamics of the HIV epidemic, two surveys were conducted in separate districts of Manipur and Nagaland in 2009: the Integrated Behavioural and Biological Assessment (IBBA) [17] and the Behavioural Tracking Survey (BTS) [16].

Elsewhere in the world, research has highlighted important differences between younger and older PWID [18], including a greater propensity to engage in risky injecting practices among younger injectors [19–23], suggesting that they are an important target group for harm reduction interventions. A high proportion of PWID in Manipur and Nagaland (i.e., >40% in most districts) are aged below 25 years [16], yet until now there has been no systematic investigation of the differences in injecting practices and sexual behaviours between the younger and older PWID in these states. This paper reports on analyses of IBBA and BTS data, and the objectives are 1) to profile differences in demographics, drug use and sexual practices across age groups and 2) to examine whether age is correlated with injecting and sexual risk behaviours. The findings from our analyses have implications for HIV prevention programmes working with PWID in these two states.

## Methods

### Study design

Data for this study was obtained from two separate cross-sectional surveys: the IBBA conducted in two districts of Manipur (Churachandpur, Bishnupur) and Nagaland (Wokha, Phek) and the BTS also conducted in two districts of Manipur (Chandel, Ukhrul) and Nagaland (Kiphere, Zunheboto). The HIV prevention programme in all eight districts was auspiced by the Bill & Melinda Gates Foundation—funded Project ORCHID. Both surveys collected information from men who inject drugs during 2009 using an interviewer-administered questionnaire and the same sampling approach (discussed below). As the BTS questionnaire was adapted from the IBBA questionnaire, many of the questions were the same, so it was feasible to combine data pertaining to variables common to both databases, incorporating all eight districts. The methods for both the IBBA and the BTS have been described in depth elsewhere [16, 17, 24, 25] and are summarized below.

Ethical approval for the IBBA was obtained in India through the Ethical Review Boards of the participating institutions: FHI360, the Regional Medical Research Centre (RMRC) in Dibrugarh, and the National AIDS Research Institute (NARI). Ethical approval for the BTS was obtained from the Institutional Review Board of the Emmanuel Hospital Association (EHA), New Delhi. Informed consent was obtained from all participants prior to administration of the behavioural questionnaire and biological testing, and confidentiality was assured.

### Sampling and participants

Respondent-driven sampling (RDS) was used to recruit participants for both the IBBA and BTS. RDS is a probability-based method for sampling from hidden populations that recruits participants using peer networks [26]. To be eligible to participate in the study, participants needed to be males aged 18 years or older and to have injected drugs for non-medical purposes at least once during the last 6 months. Peer educators with good knowledge of the local injecting drug use context were employed to screen potential participants and ensure they met the eligibility criteria.

### Data collection

Interviewer-administered questionnaires were used to collect data on demographics, drug use history and practices, sexual behaviours and participation in HIV testing. Only the IBBA participants provided blood and urine samples for HIV, HCV and STI testing. Blood samples were tested for HIV, hepatitis C (HCV) and syphilis antibodies, and urine samples were tested for *Chlamydia trachomatis* (CT). Serum samples were tested for HIV by Microelisa (J.Mitra and Company, New Delhi, DL, India), and positive tests were confirmed by Genedia HIV 1/2 ELISA 3.0 (Green Cross Life Science Corporation, Yongin, South Korea). Serum samples were tested for syphilis by rapid plasma reagin (RPR) test and confirmed by *Treponema pallidum* haemagglutination assay (TPHA). Serum samples were also tested for HCV antibody by EIA (Murex anti-HCV Version 4.0, Abbott Diagnostics, North Chicago, IL, USA). Urine samples were tested with nucleic acid amplification assay (Gen-Probe Aptima, San Diego, CA, USA) for the detection of CT.

### Sample size

For both the IBBA and BTS, a sample size of 400 in each district was estimated based on an ability to detect changes in proportions of 15% at follow-up surveys from estimated baseline values of 50% (which yield the biggest sample size), an alpha level of 0.05, a power of 90%, and a design effect of 1.5.

For the IBBA, a total of 1,650 participants (PWID) were recruited: 410 from Bishnupur, 411 from Churachandpur, 418 from Phek, and 411 from Wokha. For the BTS, a total of 1,712 participants (PWID) were recruited: 421 from Ukhrul, 415 from Chandel, 427 from Kiphire and 449 from Zunheboto. By combining the two samples, we achieved a total sample size of 3,362, which is 1% of the estimated combined total PWID population for Manipur (12,592) and Nagaland (19,959) [10, 27].

### Statistical analysis

Descriptive data obtained using RDS is typically analysed using special statistical software packages to account for the complex sampling design such as RDSAT [28, 29]. Our analyses are based on data combined from RDS surveys conducted in eight districts across Manipur and Nagaland, and it would not be legitimate to treat the data as one large RDS study because the RDS data collection coding systems were unique to each district. As such, all analyses were performed using SPSS version 21 without adjustment for the complex sampling design. Consequently, it is important to note that the results of these analyses should be viewed as if the sample were a large convenience one. The Chi-square test was used to examine differences in participant characteristics across three age groups (18–24 years, 25–34 years and 35 years or older). Binary logistic regression analyses using the forced entry method were conducted to examine whether age is correlated with unsafe injecting practices (i.e. needle/syringe sharing and drawing up drugs from common containers) and condom use with regular, casual and paid partners. Models examining the association of age with unsafe injecting behaviour were adjusted for key demographics (i.e. literacy, marital status), key drug use practices (i.e. length of injecting career, frequency of injecting, most commonly injected drug) and exposure to needle syringe programmes that disseminate HIV prevention information (i.e. usual place of procuring needles/syringes); models examining the association of age with condom use were additionally adjusted for the number of sexual partners in the past year. For all logistic regression analyses, both unadjusted and adjusted odds ratios are presented along with their respective 95% confidence intervals. Only models with acceptable diagnostics are presented; goodness of fit was assessed using the Hosmer Lemeshow test, and collinearity was assessed using the variance inflation factor (VIF). The results from the two states are reported separately because they are very different from each other both in relation to drug use patterns and HIV prevalence.

## Results

By combining the data from the IBBA and the BTS, we accumulated a sample of 1,657 PWID in Manipur and 1,705 PWID in Nagaland. In Manipur, 23.7% (*n* =392) were aged 18 to 24 years, 54.1% (*n* =897) were aged 25 to 34 years, and 22.2% (*n* =368) were aged 35 and over. In Nagaland, the participants were substantially younger; 43.6% (*n* =743) were aged 18 to 24 years, 45.9% (*n* =783) were aged 25 to 34 years, and just 10.5% (*n* =179) were aged 35 and over. The median age at first injection was 22.0 years in both Manipur and in Nagaland; the proportion of participants who first injected before the age of 20 years was 26.9% (*n* =444) in Manipur and 26.2% (*n* =443) in Nagaland.

### Demographics, drug use and sexual practices across age groups

Differences in demographics, drug use and sexual practices across age groups are profiled in Table 1. Higher proportions of younger PWID were literate and had never been married. Older PWID were more likely to have been injecting for a longer period (i.e. 3 or more years); however, a substantial proportion of PWID aged 18 to 24 years had been injecting for 3 years or more in both Manipur (35.6%) and Nagaland (28.4%). In Nagaland, younger PWID aged 18 to 24 years (65.3%) were more likely to usually obtain their new needles/syringes from NGO-delivered needle syringe programmes (NSPs) compared to PWID aged 35 years or older (40.3%). In Manipur, drawing up drugs from a common container was more prevalent behaviour in younger PWID aged 18 to 24 years (27.3%) compared to PWID aged 35 years or older (17.7%); however, there were no differences across age groups in either state regarding the proportion of PWID who had shared a needle/syringe in the past month.

**Table 1:** **Participant characteristics across age groups, by state**

	Manipur (*n*=1,657)					Nagaland (*n*=1,705)				
	18–24 years % (*n*)	25–34 years % (*n*)	35 + years % (*n*)	Total % (*n*)	*p*value ^a^	18–24 years % (*n*)	25–34 years % (*n*)	35 + years % (*n*)	Total % (*n*)	*p*value ^a^
Literacy					0.017					0.013
Illiterate	9.4 (37)	7.5 (67)	12.5 (46)	9.1 (150)		18.3 (136)	23.8 (186)	25.7 (46)	21.6 (368)	
Literate	90.6 (355)	92.5 (830)	87.5 (322)	90.9 (1,507)		81.7 (607)	76.2 (597)	74.3 (133)	78.4 (1,337)	
Marital status					<0.001					<0.001
Never married	89.5 (351)	53.6 (481)	18.8 (69)	54.4 (901)		91.9 (683)	52.9 (413)	17.4 (31)	66.2 (1,127)	
Currently married	8.2 (32)	36.6 (328)	66.6 (245)	36.5 (605)		6.9 (51)	42.6 (333)	73.0 (130)	30.2 (514)	
Widowed/divorced/separated	2.3 (9)	9.8 (88)	14.7 (54)	9.1 (151)		1.2 (9)	4.5 (35)	9.6 (17)	3.6 (61)	
Length of injecting career					<0.001					<0.001
< 3 years	64.4 (251)	20.8 (186)	13.7 (50)	29.5 (487)		71.6 (530)	33.6 (262)	21.7 (38)	49.0 (830)	
≥ 3 years	35.6 (139)	79.2 (708)	86.3 (316)	70.5 (1,163)		28.4 (210)	66.4 (517)	78.3 (137)	51.0 (864)	
Most commonly injected drug					<0.001					0.004
Heroin	87.8 (344)	96.8 (868)	99.5 (366)	95.2 (1,578)		14.9 (111)	18.8 (147)	8.4 (15)	16.0 (273)	
SP^b^	6.9 (27)	2.2 (20)	0.3 (1)	2.9 (48)		82.1 (610)	77.7 (608)	86.0 (154)	80.5 (1,372)	
Other	5.4 (21)	1.0 (9)	0.3 (1)	1.9 (31)		3.0 (22)	3.6 (28)	5.6 (10)	3.5 (60)	
Frequency of injecting					0.140					0.244
Injected less than daily	35.1 (133)	33.0 (291)	28.5 (101)	32.5 (525)		79.9 (504)	76.1(537)	78.2 (133)	77.9 (1,174)	
Injected daily	64.9 (246)	67.0 (590)	71.5 (254)	67.5 (1,090)		20.1 (127)	23.9 (169)	21.8 (37)	22.1 (333)	
Place usually procure needles/syringes					0.391					<0.001
Needle syringe programme	77.3 (303)	79.5 (713)	82.9 (305)	79.7 (1,321)		65.3 (483)	51.4 (402)	40.3 (71)	56.3 (956)	
Chemist / pharmacy	19.4 (76)	17.8 (160)	15.2 (56)	17.6 (292)		32.6 (241)	47.6 (372)	58.5 (103)	42.2 (716)	
Other	3.3 (13)	2.7 (24)	1.9 (7)	2.7 (44)		2.2 (16)	1.0 (8)	1.1 (2)	1.5 (26)	
Shared needle/syringe - past month					0.474					0.918
No	85.2 (310)	87.3 (748)	88.1 (310)	87.0 (1,368)		62.0 (426)	62.2 (423)	60.4 (90)	61.9 (939)	
Yes	14.8 (54)	12.7 (109)	11.9 (42)	13.0 (205)		38.0 (261)	37.8 (257)	39.6 (59)	38.1 (577)	
Drew drugs from common container at last injection					0.005					0.786
No	72.7 (285)	75.5 (677)	82.3 (303)	76.3 (1,265)		48.9 (363)	50.6 (396)	50.3 (90)	49.8 (849)	
Yes	27.3 (107)	24.5 (220)	17.7 (65)	23.7 (392)		51.0 (379)	49.4 (387)	49.7 (89)	50.1 (855)	
Ever had sex					<0.001					<0.001
No	25.8 (101)	9.6 (86)	4.9 (18)	12.4 (205)		14.8 (110)	3.3 (26)	0.0 (0)	8.0 (136)	
Yes	74.2 (291)	90.4 (811)	95.1 (350)	87.6 (1,452)		85.2 (632)	96.7 (757)	100.0 (179)	92.0 (1,568)	
Number of female sex partners - past year^c^					<0.001					<0.001
None	17.5 (51)	22.4 (182)	19.4 (68)	20.7 (301)		3.4 (21)	8.3 (62)	16.7 (29)	7.3 (112)	
1	32.3 (94)	51.8 (420)	69.7 (244)	52.2 (758)		30.0 (186)	42.3 (315)	50.0 (87)	38.2 (588)	
≥ 2 Partners	50.2 (146)	25.8 (209)	10.9 (38)	27.1 (393)		66.6 (413)	49.3 (367)	33.3 (58)	54.5 (838)	
Female sexual partners – past year^c^										
Regular non-paid partner(s)	44.3 (129)	56.8 (461)	72.6 (254)	58.1 (844)	<0.001	75.1 (471)	78.2 (589)	82.7 (148)	77.5 (1,208)	0.081
Casual non-paid partner(s)	47.8 (139)	21.8 (177)	9.1 (32)	24.0 (348)	<0.001	55.1 (343)	39.1 (292)	27.5 (49)	44.2 (684)	<0.001
Paid partner(s)	17.9 (52)	14.8 (120)	6.3 (22)	13.4 (194)	<0.001	5.6 (35)	5.2 (39)	8.9 (16)	5.8 (90)	0.149
Consistent condom use with different types of female partners – past year^d^										
Regular non-paid partner(s)	16.7 (21)	7.0 (32)	9.9 (25)	9.4 (78)	0.004	17.7 (82)	9.1 (53)	2.7 (4)	11.7 (139)	<0.001
Casual non-paid partner(s)	28.4 (40)	16.6 (30)	17.6 (6)	21.3(76)	0.032	38.7 (135)	27.8 (83)	16.0 (8)	32.4 (226)	<0.001
Paid partner(s)	57.7 (30)	43.3 (52)	50.0 (11)	47.9 (93)	0.219	52.8 (19)	34.1 (14)	18.8 (3)	38.7 (36)	0.049
Ever had an HIV test	31.6 (124)	58.7 (525)	62.0 (228)	53.0 (877)	0.000	37.2 (269)	46.3 (342)	43.8 (70)	42.0 (681)	0.002
Tested positive for different types of infections^e^										
HIV	5.9 (9)	27.3 (138)	65.0 (106)	30.8 (253)	0.000	0.0 (0)	2.3 (9)	2.7 (3)	1.4 (12)	0.021
Hepatitis C (HCV)	53.3 (81)	74.9 (379)	90.2 (147)	73.9 (607)	0.000	8.8 (28)	16.4 (65)	21.4 (24)	14.1 (117)	0.001
Reactive syphilis serology	2.0 (3)	4.3 (22)	4.3 (7)	3.9 (32)	0.398	7.5 (24)	16.1 (64)	20.5 (23)	13.4 (111)	<0.001
Chlamydia	2.6 (4)	2.0 (10)	0.6 (1)	1.8 (15)	0.384	12.5 (40)	7.6 (30)	4.5 (5)	9.1 (75)	0.014

^a^ *p* value based on chi-square test.

^b^Spasmoproxyvon (SP), the brand name for a synthetic opiate analgesic containing dextropropoxyphene, dicyclomine hydrochloride and paracetamol.

^c^Among sexually active participants.

^d^Reported that, in general, condoms are used every time with this type of partner. Analysis based on sub-sample of participants who reported having these types of partners in the preceding 12 months.

^e^Based on IBBA data only (Manipur *n* =821, Nagaland *n* =829), as BTS did not collect biological data.

In both states, a substantially higher proportion of PWID aged 18 to 24 years had two or more female sexual partners in the preceding 12 months (Manipur 50.2%, Nagaland 66.6%) compared to PWID aged 35 or older (Manipur 10.9%, Nagaland 33.3%). PWID of all ages reported low levels of consistent condom use with the different types of female sexual partners; however, consistent condom use with regular and casual partners was lowest among older PWID.

### Disease prevalence across age groups

Exposure to HIV testing was highest among older PWID. Not surprisingly, PWID aged 35 years or older had a much higher prevalence of HIV and HCV (Manipur, HIV 65.0%, HCV 90.2%; Nagaland, HIV 2.7%, HCV 21.4%) compared to PWID aged 18 to 24 years (Manipur, HIV 5.9%, HCV 53.3%; Nagaland, HIV 0.0%, HCV 8.8%). In Nagaland, the prevalence of reactive syphilis serology was highest among older PWID and the prevalence of chlamydia was highest among younger PWID; there were no significant differences in the prevalence of syphilis or chlamydia across age groups in Manipur.

### Association between age and needle/syringe sharing

After adjusting for confounding, the likelihood of needle/syringe sharing and drawing up drugs from a common container was highest for PWID aged 18 to 24 years in both states (Table 2). Compared to PWID aged 35 years or older, PWID aged 18 to 24 years were over one and a half times more likely to share needles/syringes in Manipur (OR =1.84) and Nagaland (OR =1.60). Compared to PWID aged 35 years or older, PWID aged 18 to 24 years were almost two times as likely to have drawn up drug solution from a common container at their last injection in Manipur (OR =1.79).

**Table 2:** **Binary logistic regression for injecting risk behaviours across age groups, by state**

Age (years)	Manipur				Nagaland			
	Sharing needles/syringes in past month		Drew up drugs from common container at last injection		Sharing needles/syringes in past month		Drew up drugs from common container at last injection	
	Unadjusted OR (95% CI) (*n*=1,558)	Adjusted OR ^a^ (95% CI) (*n*=1,537)	Unadjusted OR (95% CI) (*n*=1,657)	Adjusted OR ^a^ (95% CI) (*n*=1,608)	Unadjusted OR (95% CI) (*n*=1,577)	Adjusted OR ^a^ (95% CI) (*n*=1,362)	Unadjusted OR (95% CI) (*n*=1,704)	Adjusted OR ^a^ (95% CI) (*n*=1,487)
18–24	1.20 (0.78–1.82)	1.84 (1.10–3.08)*	1.75 (1.24–2.48)**	1.79 (1.17–2.73)**	0.89 (0.63–1.28)	1.60 (1.01–2.53)*	1.06 (0.76–1.46)	1.49 (0.98–2.26)
25–34	1.05 (0.73–1.51)	1.12 (0.76–1.65)	1.52 (1.11–2.06)**	1.35 (0.97–1.87)	0.94 (0.66–1.33)	0.98 (0.65–1.48)	0.94 (0.71–1.37)	1.33 (0.74–1.53)
35+	1	1	1	1	1	1	1	1

*p <0.05; **p <0.01; ***p <0.001.

^a^Adjusted for literacy, marital status, length of injecting career, frequency of injecting, most commonly injected drug, and usual place for procuring new needles/syringes.

### Association between age and condom use

The binary logistic regression model for consistent condom use with different partners is presented in Table 3. In Nagaland, PWID aged 18 to 24 years were more likely to use condoms consistently with both casual (OR =3.12) and paid (OR =17.65) female sexual partners compared to PWID aged 35 years or older. In Manipur, there were no statistically significant differences in the likelihood of consistently using condoms with any partner between PWID aged 18 to 24 and those aged 35 years or older.

**Table 3:** **Binary logistic regression for consistent condom use in the past year across age groups, by state**

Age (years)	Manipur						Nagaland					
	Consistent condom use with regular partners ^a^		Consistent condom use with casual partners ^a^		Consistent condom use with paid partners ^a^		Consistent condom use with regular partners ^a^		Consistent condom use with casual partners ^a^		Consistent condom use with paid partners ^a^	
	Unadjusted OR (95% CI) (*n*=832)	Adjusted OR ^b^ (95% CI) (*n*=827)	Unadjusted OR (95% CI) (*n*=356)	Adjusted OR ^b^ (95% CI) (*n*=355)	Unadjusted OR (95% CI) (*n*=194)	Adjusted OR ^b^ (95%CI) (*n*=193)	Unadjusted OR (95% CI) (*n*=1,193)	Adjusted OR ^b^ (95%CI) (*n*=1,163)	Unadjusted OR (95% CI) (*n*=698)	Adjusted OR ^b^ (95% CI) (*n*=682)	Unadjusted OR (95% CI) (*n*=93)	Adjusted OR ^b^ (95% CI) (*n*=89)
18–24	1.82 (0.97–3.39)	0.47 (0.19–1.16)	1.85 (0.71–4.80)	1.23 (0.37–4.05)	1.36 (0.50–3.71)	0.72 (0.22–2.31)	7.62 (2.74–21.17)***	1.20 (0.33–4.40)	3.31 (1.51–7.27)**	3.12 (1.05–9.27)*	4.84 (1.18–19.95)*	17.65 (1.42–219.16)*
25–34	0.69 (0.40–1.19)	0.34 (0.17–0.65)**	0.93 (0.35–2.42)	0.95 (0.31–2.90)	0.77 (0.31–1.90)	0.56 (0.20–1.55)	3.55 (1.26–9.97)**	1.46 (0.42–5.10)	2.02 (0.91–4.48)	1.95 (0.69–5.53)	2.25 (0.55–9.22)	5.53 (0.57–53.41)
35+	1	1	1	1	1	1	1	1	1	1	1	1

*p <0.05; **p <0.01; ***p <0.001.

^a^Reported that, in general, condoms are used every time with this type of partner; the other options (most of the times, sometimes, and never) were combined to construct a binary variable. Only participants with these types of sexual partners over the past year were included in the analyses.

^b^Adjusted for literacy, marital status, length of injecting career, frequency of injecting, usual place for procuring new needles/syringes and number of female sex partners.

## Discussion

An important aspect of the demographic profile of PWID in Manipur and Nagaland presented in this paper is that a substantial proportion of the adult PWID participants were aged 18 to 24 years in both states (Manipur =23.7%, Nagaland = 43.6%). Furthermore, in both Manipur and Nagaland, one in four PWID first injected illicit drugs before the age of 20 years, indicating that many are entering into injecting drug use at a young age. The analyses presented in this paper provide a greater understanding of the differences between younger and older PWID so that HIV prevention programmes can be targeted accordingly.

Risky injecting and sexual behaviours were common among PWID in Manipur and Nagaland, coupled with a high prevalence of HIV, hepatitis C and STIs. Interestingly, our multivariate analyses reveal important and somewhat conflicting differences between PWID across age groups with regard to risky injecting practices and sexual behaviours. After adjusting for a range of important confounders, younger PWID in both Manipur and Nagaland were more likely to share needles/syringes, and younger PWID in Manipur were also more likely to draw up drug solutions from common containers, creating opportunities for HIV and HCV transmission. Yet by contrast, younger PWID in Nagaland were more likely to consistently use condoms with both casual and paid female partners than older PWID.

Our finding that younger PWID are more likely to engage in risky injecting behaviours has also been observed in Delhi [30] and is not unique to this region of the world. A high level of risky injecting behaviours has been observed among younger injectors in Australia, Brazil, Afghanistan and the United States [19–21, 23]. A recently published survey of almost 7,000 PWID across several major cities in Australia found that after adjusting for length of injecting career, each 5-year increase in age was associated with an average 16% reduction in the likelihood of receptive needle sharing [20]. One plausible explanation for such results is that younger PWID have poorer knowledge of safe injecting practices and/or have less access to clean injecting equipment. Another explanation is that young adulthood is thought to be a time of experimentation and greater risk-taking and that young adults are more vulnerable to peer influences that encourage risky behaviour [31, 32].

The local socio-cultural and political context for young men in Manipur and Nagaland, a region with a rich tribal history, is also relevant to understanding the behaviours of younger PWID. Masculinity in the northeast is influenced by a convergence of the traditional/tribal male warrior role, with ethno-nationalism and armed struggle against the Indian state and between different ethnic and tribal groups [33]. Research on initiation into injecting drug use among PWID in Manipur and Nagaland found that injecting among young men was closely connected with the influence of peers and social networking, as well as notions of masculinity and a right of passage that bonds young men with their friends [34, 35]. If we adopt a youth and equity lens [36, 37], HIV prevention interventions in Manipur and Nagaland should include interventions tailored for young people, such that: 1) young people have access to resources (i.e. information and equipment such as needles/syringes), 2) drug services are "youth-friendly", i.e. they engage with and respond to the information and treatment needs of young people, 3) information and education on drug use is widely available to adolescents and 4) opportunities to interrupt the transition to injecting drug use (e.g. life skills programmes) are explored.

Our finding that younger PWID in Nagaland were significantly more likely to consistently use condoms with casual and paid female partners is a striking reversal of the association between age and injecting risks. A study among men attending sexually transmitted disease clinics in Pune, India, similarly found that younger men were more likely to use condoms than older men [38]. The authors hypothesized that younger men have been exposed to broader community HIV/STI awareness programmes that promoted condom use, whereas older men were unlikely to have benefitted from these education efforts. This is probably true in the socially conservative context of Northeast India as well, where it is only over the last decade or so that HIV/STI awareness programmes have begun to widely promote the use of condoms. Furthermore, research conducted among men in Chennai, South India, found that there was a need to develop a "condom habit" from a young age, as using condoms at first sexual experience was a strong predictor of future condom use [39]. A different set of public health strategies may be needed in order to reach older men with safe sex messages, particularly older PWID, many of whom may have ingrained attitudes and behaviours regarding condom use.

### Limitations

Our findings are subject to some limitations. Reporting of certain behaviours may have been influenced by recall and social desirability bias resulting in some risk behaviours being under-reported. As with all cross-sectional surveys, causal relationships cannot be firmly determined. All IBBA and BTS survey participants were recruited using RDS and such data are typically analysed using RDSAT to account for the complex sampling design. We have combined data collected across eight districts for the IBBA and BTS surveys to construct a large convenience sample (*n* = 3,362) and have consequently been unable to account for the complex sampling design in our analyses. The limitation of our approach is that our analyses are essentially based on a convenience sample with restricted generalisability of the observed associations. Finally, the participants were sampled through peer networks for the IBBA and BTS surveys, and it is unknown how deep this approach penetrated the sub-population of PWID in these districts; it is possible that PWID who were not recruited had different characteristics to the study participants.

## Conclusion

The analyses presented in this paper highlight important differences in needle/syringe sharing and condom use across age groups among PWID in Manipur and Nagaland, Northeast India. Younger PWID were more likely to share needles and syringes, and older PWID in Nagaland were less likely to consistently use condoms with casual and paid female partners. There is a clear need to promote different harm reduction strategies across age groups to ensure that younger PWID are more effectively reached with safe injecting messages and equipment and safe sex messages and condoms are effectively delivered to older PWID.
